# Draft Genome Sequence of Talaromyces cellulolyticus Strain Y-94, a Source of Lignocellulosic Biomass-Degrading Enzymes

**DOI:** 10.1128/genomeA.00014-15

**Published:** 2015-02-26

**Authors:** Tatsuya Fujii, Hideaki Koike, Shigeki Sawayama, Shinichi Yano, Hiroyuki Inoue

**Affiliations:** a Biomass Refinery Research Center, National Institute of Advanced Industrial Science and Technology (AIST), Higashi-Hiroshima, Hiroshima, Japan; b Bioproduction Research Institute, National Institute of Advanced Industrial Science and Technology (AIST), Tsukuba, Ibaraki, Japan; c Division of Applied Biosciences, Graduate School of Agriculture, Kyoto University, Sakyo-ku, Kyoto, Japan

## Abstract

Talaromyces cellulolyticus (formerly Acremonium cellulolyticus) is a promising fungus for cellulase production. Here, we present the draft genome sequence of T. cellulolyticus strain Y-94. The genome is 36.4 Mbp long and contains genes for several enzymes involved in the degradation of lignocellulosic biomass, including cellulases, hemicellulases, pectinases, and amylases.

## GENOME ANNOUNCEMENT

Talaromyces cellulolyticus strain Y-94 (CBS 136886, FERM BP-5826), which was isolated in Japan (1), is one of several promising filamentous fungi for the industrial production of cellulase and hemicellulase to hydrolyze lignocellulosic biomass (2–5). The taxonomic classification of this organism has been revised from the genus Acremonium to Talaromyces (6). Recently, a transformation system with a uracil auxotrophic strain has been developed, and some glycoside hydrolase (GH) family enzymes and transcription factors have been analyzed (7–11).

We present here the draft genome sequence of T. cellulolyticus Y-94. 454/Roche (FLX Titanium) and Illumina Genome Analyzer II sequencers were used in this study. T. cellulolyticus Y-94 genomic DNA was sheared into 3-kb paired-end fragments to generate a library according to the Roche paired-end library preparation method manual. A 454 Titanium draft library with 699 Mb of total reads and an average read length of 391 bases, which provided 19-fold coverage of the genome, was generated. Reads were assembled using the gsAssembler (454 Life Sciences, Roche Applied Science, Branford, CT, USA), and 60 scaffolds were obtained. The genome size of T. cellulolyticus predicted by the total scaffold length was 36.4 Mbp. Illumina Genome Analyzer II sequencing was done to account for 454/Roche sequencing errors. T. cellulolyticus genomic DNA was sheared into 0.3-kb fragments for paired-end library construction. An Illumina draft library with 2,686 Mb of total reads and a read length of 75 bases was generated. Reads were mapped onto the 454 assembly using Mapping and Assembly with Quality (Maq) and used to correct 454 sequencing errors. In total, 10,980 open reading frames (ORFs) were predicted using AUGUSTUS (12) trained on Aspergillus nidulans FGSC A4 gene models.

We screened the draft sequence for GH family genes. At least 249 ORFs were annotated as GH family proteins, including 133 potentially secreted proteins (based on a SignalP version 4.1 analysis and our secretome data). The number of GH family genes of T. cellulolyticus was similar to that of A. nidulans (247 genes), less than that of Aspergillus oryzae (285 genes), and greater than that of Trichoderma reesei (200 genes) (13). Based on the CAZy database (14), carbohydrate-active enzymes related to the hydrolysis of lignocellulosic biomass were identified: 22 cellulases (12 GH5s [including hemicellulases such as mannanase], 1 GH6, 2 GH7s, 4 GH12s, 1 GH61, and 2 GH45s); 37 hemicellulases (22 GH43s, 1 GH10, 7 GH11s, 1 GH74, 1 GH62, 1 GH53, 1 GH54, 2 GH67s, and 1 GH26); 38 pectinases (16 GH28s, 12 GH78s, 4 PL1s, 2 PL4s, 2 CE8s, and 2 CE12s); and 8 amylases (5 GH13s and 3 GH15s). These results support the ability of T. cellulolyticus to degrade various types of biomass (1, 3, 10, 15). The genome sequence data will provide suggestions for improvements in cellulase and hemicellulase production in T. cellulolyticus.

### Nucleotide sequence accession numbers.

The nucleotide sequence of T. cellulolyticus strain Y-94 has been deposited at DDBJ/EMBL/GenBank as follows: 1,723 contigs under accession numbers BBPS01000001 to BBPS01001723 and 60 scaffolds under accession numbers DF933797 to DF933856. The version described in this study is the first version.
